# Chronische Rhinosinusitis mit Polyposis nasi: Retrospektive Analyse des therapeutischen Vorgehens bei 463 Patienten

**DOI:** 10.1007/s00106-024-01479-y

**Published:** 2024-05-03

**Authors:** J. Strauss, R. Lochbaum, T. K. Hoffmann, B. Mayer, H. Appel, J. Hahn

**Affiliations:** 1 Klinik für Hals-Nasen-Ohrenheilkunde, Kopf- und Halschirurgie, Universitätsklinik Ulm, Frauensteige 12, 89075 Ulm, Deutschland; 2 Institut für Epidemiologie und Medizinische Biometrie, Universität Ulm, Ulm, Deutschland

**Keywords:** Asthma bronchiale, Allergische Rhinitis, Biologika, Desaktivierung, Antikörper, Asthma, Allergic rhinitis, Biologicals, Desensitization, Antibodies

## Abstract

**Hintergrund:**

Die chronische Rhinosinusitis mit Polyposis nasi (CRSwNP) wird zunehmend als multifaktorielle inflammatorische Erkrankung verstanden, deren Therapieprinzipien in den vergangenen Jahren größeren Veränderungen unterlagen. Neben operativen Maßnahmen werden topische und systemische Steroide sowie eine adaptive Acetylsalicylsäure(ASS)-Desaktivierung bei entsprechenden Indikationskriterien eingesetzt. Seit 2019 ergänzen 3 spezifische Antikörper das therapeutische Portfolio.

**Methoden:**

Es erfolgte eine retrospektive Auswertung aller Patienten, die in den Jahren 2007 und 2008 (Kollektiv A) sowie 2017 und 2018 (Kollektiv B) erstmalig aufgrund einer CRSwNP ambulant vorstellig wurden, bis inklusive Juni 2023.

**Ergebnisse:**

Der klinische Verlauf von 463 Patienten (Durchschnittsalter: 49,1 Jahre; Spannbreite: 5–82 Jahre, 65,9 % männlich) wurde in die Auswertung eingeschlossen. Eine vor Erstvorstellung begonnene konservative Behandlung mit nasalen Kortikosteroiden wurde in Kollektiv B häufiger durchgeführt (Kollektiv A 43,9 % vs. Kollektiv B 72,2 %). Bei 278 der 463 Patienten (60 %; A: 62 %, B: 58 %) erfolgte nach Erstvorstellung mindestens eine Nasennebenhöhlenoperation. Bei 101 Patienten (36,3 %) davon kam es nach einer Operation im weiteren Verlauf im Mittel nach 2,4 Jahren zu einem behandlungsbedürftigen Polyposisrezidiv. Die Indikation zur ASS-Provokation/-Desaktivierung wurde im Kollektiv B seltener gestellt, nicht zuletzt aufgrund der hohen Abbruchrate (mindestens 38 %) der Erhaltungstherapie. Bei 16 Patienten der Gesamtkohorte (3,5 %, A: *n* = 8, B: *n* = 8) war bei Auftreten des Rezidivs inzwischen die Einstellung auf eine Therapie mit einem Antikörper erfolgt.

**Schlussfolgerung:**

Ein stufenweises leitlinienkonformes Vorgehen ist sinnvoll. Der systemische Einsatz von Antikörpern bei therapieresistenten CRSwNP-Verläufen stellt eine verhältnismäßig neue Behandlungsoption dar, welche die relativ nebenwirkungsreiche und compliancearme ASS-Desaktivierung reduzieren wird.

## Abstract (en)

**Background:**

Chronic rhinosinusitis with nasal polyps (CRSwNP) is a multifactorial inflammatory disease, the treatment of which has undergone significant changes in recent years. In addition to surgical approaches, topical and systemic steroids, and adaptive acetylsalicylic acid (ASA) desensitization, three specific antibodies have complemented the therapeutic portfolio since 2019.

**Methods:**

A retrospective evaluation of all patients who presented as outpatients for the first time due to CRSwNP in 2007 and 2008 (collective A) and 2017 and 2018 (collective B) was performed, up to and including June 2023.

**Results:**

The clinical courses of 463 patients (mean age 49.1 years, range 5–82 years; 65.9% male) were included in the analysis. Conservative treatment with nasal corticosteroids started before initial presentation was more frequent in collective B (collective A 43.9% vs. collective B 72.2%). In 278 of the 463 patients (60%; A: 62%, B: 58%), at least one operation on the nasal sinuses had been performed after initial presentation; in 101 of these patients (36.3%) recurrent polyposis (within mean follow-up of 2.4 years) required further treatment. The indication for ASA provocation/desensitization was applied less frequently in collective B, also due to a high discontinuation rate (at least 38%) of the maintenance therapy. Of the total cohort, 16 patients (3.5%; A: *n* = 8, B: *n* = 8) were meanwhile switched to antibody therapy at recurrence.

**Conclusion:**

A step-by-step guideline-orientated approach is recommended in the treatment of CRSwNP. Systemic antibodies as an add-on to nasal corticosteroids are a relatively new therapeutic option for treatment-refractory CRSwNP, which reduces the indication for ASA desensitization, which is associated with a relatively high incidence of side effects and poor compliance.

Das Therapieregime der chronischen Rhinosinusitis (CRS) mit Polyposis nasi (CRSwNP) hat relevante Veränderungen erlebt. Bei der vorrangig auf einer Typ-2-Inflammation basierenden Erkrankung bestehen als langjährige Therapieoptionen Steroide sowie operative Maßnahmen. Die adaptive Acetylsalicylsäure(ASS)-Desaktivierung wurde über Jahre bei bestimmten Patienten empfohlen. In der sich aktuell in Überarbeitung befindlichen S2k-Leitlinie der Arbeitsgemeinschaft der Wissenschaftlichen Medizinischen Fachgesellschaften (AWMF) „Rhinosinusitis“ von 2017 wurden Antikörper als Therapiemöglichkeit im Einzelfall erwähnt. Inzwischen gibt es in Deutschland 3 zugelassene Antikörper für die Therapie der CRSwNP, die Durchführung der adaptiven ASS-Desaktivierung wird zunehmend als obsolet betrachtet. Das Kapitel „Therapie der chronischen Rhinosinusitis mit Polyposis nasi mit monoklonalen Antikörpern (Biologika)“ der S2k-Leitlinie wurde 2023 aktualisiert.

## Häufigkeit und Leidensdruck

Die chronische Rhinosinusitis mit Polyposis nasi (CRSwNP) ist ein verbreitetes Krankheitsbild mit hohem Leidensdruck. Die CRSwNP stellt eine multifaktorielle, entzündliche Erkrankung der Schleimhäute von Nase und Nasennebenhöhlen (NNH) dar, phänotypisch gekennzeichnet durch die Ausbildung von Polypen. Die genaue Ätiologie erscheint multifaktoriell – genetische und umweltbedingte Faktoren werden diskutiert, die mit der Epithelbarriere der oberen Atemwege interagieren und Entzündungswege auslösen [24]. Der CRSwNP liegt pathophysiologisch vorwiegend eine sog. Typ-2-Entzündung zugrunde, wobei auch „Mischtypen“ mit anderen Inflammationen bestehen. Deren Einfluss auf das Therapieansprechen ist Gegenstand aktueller Forschung [10]. Die Typ-2-Entzündung ist u. a. durch eine eosinophile Gewebeinfiltration, erhöhte Werte von Interleukin(IL)-5 und anderen Typ-2-assoziierten Entzündungsmediatoren wie IL‑4, IL-13, IL-25 und IL-33 gekennzeichnet [2].

**Abb. 1:**
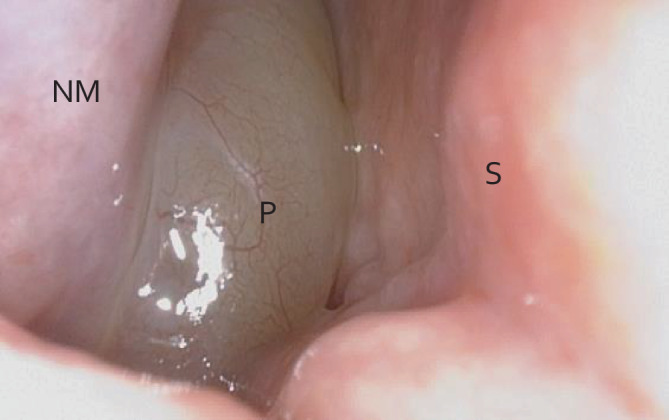
Endoskopische Darstellung einer verlegenden Polyposis nasi; *NM* untere Nasenmuschel, *S* Septum, *P* Polyp

Überwiegend tritt die CRSwNP als Erkrankung des mittleren Alters auf, die Diagnose wird bei den meisten Patienten zwischen dem 40. und 60. Lebensjahr gestellt [22]. Mittels Patientenfragebögen wird in Publikationen eine Prävalenz der CRSwNP von circa 1–4 % angegeben, je nach Region und Subgruppen (z. B. Alter) der Befragten [8]. Obwohl die Inzidenz der Erkrankung unter Frauen geringer ist, sind sie tendenziell schwerer betroffen als männliche Patienten [21]. Patienten mit CRSwNP weisen im Durchschnitt ausgeprägtere Befunde in der Computertomographie (CT) und in der Nasenendoskopie auf, verglichen mit Patienten mit chronischer Rhinosinusitis ohne Polyposis nasi [26]. Die Beschwerden der CRSwNP dauern definitionsgemäß länger als 12 Wochen an und bestehen in typischen Symptomen wie einer Nasenatmungsbehinderung, Geschmacks- und Geruchsveränderung/-minderung, nasaler Sekretion und Gesichtsschmerz [9]. Die Befunde müssen durch einen pathologischen Befund in der Rhinoskopie bzw. nasalen Endoskopie (Abb. 1) und/oder in einem bildgebenden Verfahren der NNH (zumeist digitale Volumentomographie, DVT, oder CT) bestätigt werden. Mit der Chronifizierung der Erkrankung sind häufig Epithelschädigungen und Gewebsdestruktionen verbunden, die zudem das Risiko für Virusinfektionen erhöhen [22]. Die Rezidivgefahr bei Patienten mit CRSwNP ist hoch. Nach einer Untersuchung von Lou et al. wird ein Gewebe-Eosinophilenanteil von > 27 % der Gesamtzellen oder eine absolute Gewebe-Eosinophilenzahl von > 55 als zuverlässiger prognostischer Indikator für das Wiederauftreten von Nasenpolypen innerhalb von 2 Jahren nach der Operation gesehen [15]. Zu häufigen Komorbiditäten gehören das Asthma bronchiale, Allergien (inklusive der allergischen Rhinitis), das Non-Steroidal-Anti-Inflammatory-Drugs(NSAID)-Intoleranz-Syndrom und Urtikaria [8, 12].

## Historische Entwicklung der Therapiekonzepte

Die Therapiekonzepte der CRSwNP basierten in den vergangenen Jahrzehnten auf verschiedenen Ansatzpunkten und Empfehlungen, ein neuer Meilenstein in der Therapie wurde durch die Zulassung von Biologika ab Ende 2019 erreicht. Anhand einzelner Veröffentlichungen wird hier eine kurze Übersicht über verschiedene therapeutische Ansatzpunkte gegeben.

In einer Publikation von 1969 gibt G. Brown eine Übersicht über damals angewendete Therapien bei 313 Patienten mit CRSwNP, welche aus lokalen und systemischen abschwellenden Medikamenten, lokalen und systemischen Steroiden und operativen Maßnahmen bestanden. Darüber hinaus wurde in einigen Fällen eine allergenspezifische Immuntherapie als Therapieversuch der CRSwNP durchgeführt. Diskutiert wird in der Publikation auch das erforderliche Ausmaß einer Operation: Als eine Ursache einer Rezidivpolyposis nach operativer Maßnahme wird das nicht vollständige Entfernen der polypös veränderten Schleimhaut genannt [6]. In Publikationen aus verschiedenen Jahrzehnten wird die therapeutische Rolle von lokaler und systemischer Antibiotikagabe diskutiert [4]. Gemäß der Leitlinie der Arbeitsgemeinschaft der Wissenschaftlichen Medizinischen Fachgesellschaften (AWMF) von 2017 wird von der topischen Antibiotikagabe allgemein abgeraten, die Indikation zur systemischen Gabe und empfohlene Präparate bei CRS-Patienten werden genau definiert [25]. Dekongestiva sollten nach heutigem Stand in der Therapie der chronischen Rhinosinusitis keine Rolle mehr spielen. Der Einsatz einer allergologischen Testung wird in den Leitlinien definiert [25].

Ein wesentlicher historischer Beitrag in der Therapie der CRSwNP war die Etablierung der endoskopisch gestützten Operationstechnik (Functional Endoscopic Sinus Surgery, FESS): Die endoskopische NNH-Chirurgie wurde 1985 u. a. von Wigand, Stammberger und Kennedy geprägt und in den klinisch-chirurgischen Alltag eingeführt [20]. Diese ist zum operativ empfohlenen Standard geworden. Die Indikation zur Operation wird in aller Regel erst bei Versagen der konservativen Therapie gestellt [25].

Vor allem bei Patienten mit der Symptomtrias einer CRSwNP, einem nichtallergischen Asthma bronchiale und einer NSAID-Intoleranz („NSAID exacerbated respiratory disease“; NERD) besteht eine signifikant höhere Rate von Rezidivpolyposis nach operativen Maßnahmen [1]. Als „kausalen Therapieansatz“ wurde schon vor über 100 Jahren das Konzept der adaptiven Acetylsalicylsäure(ASS)-Desaktivierung aufbauend auf Publikationen von Samter und Widal um 1922 schrittweise u. a. von Zeiss, Lockey, Stevenson et al. entwickelt. Nach erfolgter Desaktivierung durch Überschreiten einer ASS-Schwellendosis sollen CRSwNP-Patienten mit nachgewiesener NSAID-Intoleranz durch eine Toleranzentwicklung keine Symptome nach ASS-Einnahme mehr entwickeln. Eine Dauereinnahme einer höheren Dosis ASS (circa 300–1000 mg, je nach Publikation) wird konsekutiv empfohlen, um so eine klinische Besserung der Symptome zu erzielen [23].

Seit dem Jahr 2019 sind in Deutschland schrittweise 3 monoklonale Antikörper für die Indikation „Zusatztherapie zu intranasalen Glukokortikosteroiden für die Behandlung Erwachsener mit schwerer chronischer Rhinosinusitis mit Nasenpolypen“ zugelassen worden [19]: Dupilumab (hemmt den IL4-/IL-13-Signalweg), Omalizumab (bindet an Immunglobulin E, IgE, und verhindert damit die Bindung von IgE an den Rezeptor) und Mepolizumab (bindet an IL-5). Es besteht seither eine zunehmende Evidenz zur Therapie mit diesen Präparaten bei CRSwNP-Patienten, die unter bisherigen Therapiekonzepten keine ausreichende Therapiekontrolle erfuhren.

## Aktuelle Therapieempfehlungen und Leitlinienkonzepte

- Die S2k-Leitlinie „Rhinosinusitis“ der Deutschen Gesellschaft für Hals-Nasen-Ohren-Heilkunde, Kopf- und Hals-Chirurgie (DGHNO-KHC) und der Deutschen Gesellschaft für Allgemeinmedizin und Familienmedizin (DEGAM), 2017 [25]
- Das European Position Paper on Rhinosinusitis and Nasal Polyps, 2020 [9]
- Das Positionspapier: Anwendung von Biologika bei chronischer Rhinosinusitis mit Polyposis nasi (CRSwNP) im deutschen Gesundheitssystem – Empfehlungen des Ärzteverbandes Deutscher Allergologen (AeDA) und der AGs Klinische Immunologie, Allergologie und Umweltmedizin und Rhinologie und Rhinochirurgie der Deutschen Gesellschaft für HNO-Heilkunde, Kopf- und Halschirurgie (DGHNOKHC), 2020 [13]
- Das „EUFOREA expert board meeting on uncontrolled severe chronic rhinosinusitis with nasal polyps (CRSwNP) and biologics: Definitions and management“, 2021 [3]
- Die Therapie der chronischen Rhinosinusitis mit Polyposis nasi (CRScNP) mit monoklonalen Antikörpern (Biologika): S2k-Leitlinie der DGHNO-KHC und DEGAM, 2023 [19]

Im Folgenden wurden zentrale Empfehlungen der 5 genannten Leitlinien und Positionspapiere zur Therapie der CRSwNP zusammengestellt:

- Die aktuelle Studienlage befürwortet die langfristige Anwendung von nasalen Kortikosteroiden und zeigt sie als wirksam und sicher.
- Nasenspülung mit isotonischer Kochsalzlösung oder Ringerlösung ist eine wirksame Behandlung bei CRS-Patienten.
- Nach erfolgter FESS kann die regelmäßige Einnahme von nasalen Kortikosteroiden das Wiederauftreten von Polypen verhindern/verzögern.
- 1–2 Zyklen „Stoßtherapie“ systemischer Kortikosteroide pro Jahr sind eine mögliche sinnvolle Zusatztherapie bei CRSwNP-Patienten mit teilweise oder unkontrollierter Erkrankung.
- Die Entscheidung zur Operation sollte nur bei symptomatischen CRSwNP-Patienten erfolgen, bei unzureichendem Ansprechen auf eine konservative Behandlung.
- Biologika sollen bei erwachsenen Patienten mit schwerer CRSwNP bei fehlender Krankheitskontrolle als Zusatztherapie zu intranasalen Kortikosteroiden erwogen werden, wobei präparatespezifische Zulassungskriterien zu beachten sind.
- Aufgrund des Wirtschaftlichkeitsgebots für Verordnungen im deutschen GKV-System (gesetzliche Krankenversicherung) muss die Verordnung von Biologika ausreichend, zweckmäßig, und notwendig erscheinen.
- Eine orale ASS-Desaktivierung mit Langzeiteinnahme von ASS kann eine Behandlung für Patienten mit CRSwNP und NSAID-Intoleranzsyndrom sein, unter Berücksichtigung der Patientencompliance und des Nebenwirkungsprofils des ASS.

### Ziel der vorliegenden Arbeit.

Auf Basis der beschriebenen Veränderungen im therapeutischen Vorgehen der CRSwNP entstand das Konzept der vorliegenden Arbeit. Eine weitere Grundlage war, dass die Autoren auf subjektiver Ebene eine Änderung der Patientencharakteristika wahrnahmen (z. B. jüngeres Patientenalter bei der Erstvorstellung). Ziel der Arbeit war es, retrospektiv bei 2 Patientenkollektiven mit einem unterschiedlichem Beobachtungszeitraum Patientencharakteristika herauszuarbeiten und die therapeutischen Schritte auszuwerten. Hierzu sollte eine primär deskriptive Analyse der durchgeführten Therapien und des Therapieansprechens (z. B. Therapie vor Vorstellung in der Universitätsklinik, Anzahl erforderlicher Operationen, ASS-Desaktivierung, Antikörpertherapie) erfolgen. Aufgrund der Differenz des Nachbeobachtungszeitraums ist eine separate und rein deskriptive Betrachtung der Kollektive erforderlich und kein direkter Vergleich möglich.

## Studiendesign und Methodik

Es erfolgte eine retrospektive Analyse mithilfe der hausinternen elektronischen Patientenakte (ePA). Ausgewertet und in die Studie eingeschlossen wurden alle Patienten (keine Einschränkung hinsichtlich Alter und Geschlecht), die in den Jahren 2007, 2008, 2017 und 2018 eine Erstvorstellung aufgrund einer CRSwNP in der HNO-Hochschulambulanz der Autoren hatten. Die Jahre wurden aus den folgenden Gründen gewählt: Im Jahr 2006 wurde die ePA in der HNO-Klinik eingeführt, sodass davon auszugehen war, dass sich im Jahr 2007 schon eine routinierte Dokumentation etabliert hatte. Die CRSwNP-Patienten der Jahre 2007 und 2008 (in der Auswertung als Kollektiv A bezeichnet) wurden in Bezug auf ihren klinischen Charakteristika und dem weiteren klinischen Verlauf ausgewertet, ebenso wie Patienten, die aufgrund einer CRSwNP in der HNO-Hochschulambulanz in den Jahren 2017 und 2018 (in der Auswertung als Kollektiv B bezeichnet) vorstellig wurden. Die Jahre 2017 und 2018 wurden gewählt, um einen Zeitraum mit klarer Differenz (10 Jahre) zu der Kohorte 2007 und 2008 herauszuarbeiten und dennoch schon einen klinischen Verlauf bis zum Auswertungszeitraum über mehrere Jahre vorliegen zu haben. Die Auswertung umfasste den Zeitraum bis inklusive Juni 2023. Darüber hinaus ausgewertet wurden die zuweisende Fachdisziplin und die schon vor der Vorstellung in der Universitätsambulanz erfolgten Therapien. Schwerpunkt lag im Gesamten auf dem therapeutischen Vorgehen. Von Kollektiv A konnte bis zum Auswertungszeitpunkt eine Periode von 15 bzw. 16 Jahren ausgewertet werden. Es wurde analysiert, wie häufig es zu einer ambulanten bzw. stationären Wiedervorstellung kam, wie viele Patienten mit einer rein konservativen Therapie ausreichend behandelt waren, bei wie vielen eine Operation stattfinden musste und der Anteil von Patienten mit erforderlichen mehrfachen NNH-Operationen. Unter anderem aufgrund des weitläufigen Einzugsgebiets und der postoperativen Tamponade wurden die ausgewerteten NNH-Operationen in der Universitätsklinik der Autoren mit postoperativer stationärer Überwachung durchgeführt. Ebenso wurde der Anteil an Patienten erfasst, welcher letztendlich eine Antikörpertherapie erhielt und/oder eine ASS-Desaktivierung. Von Kollektiv B lag ein Zeitraum von 5 bzw. 6 Jahren vor, der hinsichtlich der klinischen Verläufe und durchgeführten Therapien bis heute ausgewertet werden konnte. Weitere Parameter, die erhoben wurden, sind das Vorliegen einer Riechstörung und der nasale Polypenscore (NPS), wenn retrospektiv möglich. Bei allen Patienten lag präoperativ eine Schichtbildgebung vor.

Die adaptive ASS Desaktivierung erfolgte nach klinikinternem Standard mit einer oralen, einfach verblindeten, placebokontrollierten Provokation von 6–500 mg ASS und bei entsprechender Reaktion mit einer „Erhaltungstherapie“ mit 300 mg ASS protect einmal täglich.

Die Patienten wurden über die Klinik-Software SAP ermittelt, indem nach Patienten mit dem Diagnosecode der CRSwNP (J.33) mit ambulanter Erstvorstellung in den Jahren 2007, 2008, 2017 und 2018 in der Hochschulambulanz der HNO-Universitätsklinik gesucht wurde. Nicht ausgewertet wurden Patienten, bei denen der Diagnosecode nicht korrekt verwendet wurde, oder Patienten, die primär aufgrund einer anderen Diagnose vorstellig waren und behandelt wurden, sodass die Diagnose J.33 CRSwNP nur eine Nebendiagnose war und der Therapieverlauf nicht ermittelt werden konnte.

Wenn Zahlen in der Publikation von der Anzahl der Gesamtkohorte abweichen, liegt die Differenz darin begründet, dass bei einzelnen Patienten zu der Fragestellung in der retrospektiven Auswertung keine Angabe vorlag. Aufgrund der unterschiedlich vergleichenden Analysezeiträume wurde eine primär deskriptive Auswertung durchgeführt.

Vor dem Forschungsvorhaben wurde das Projekt mit einem positiven Votum der Ethikkommission der Universität Ulm bescheinigt (Nr. 321/22), ohne Erfordernis des schriftlichen Einverständnisses der Patienten aufgrund des retrospektiven Settings.

## Ergebnisse

### Allgemeine Patientencharakteristika

Insgesamt wurden 463 Patientenverläufe mit CRSwNP ausgewertet. In den Jahren 2007/08 wurden 251 Patienten (Kollektiv A) erstmalig in der Hochschulambulanz vorstellig, 212 Patienten in den Jahren 2017/18 (Kollektiv B). Das Alter der Patienten bei Erstvorstellung reichte von 5–82 Jahre (Mittelwert: 49,1 Jahre). Der Mittelwert der Patienten aus Kollektiv A lag bei 50,1 Jahren, aus Kollektiv B bei 48 Jahren. Insgesamt waren 305 Patienten (65,9 %) männlich, 157 (33,9 %) weiblich und ein Patient divers. Zum Zeitpunkt der Erstvorstellung waren 11 der 463 CRSwNP-Patienten (2,4 %) minderjährig (5 weiblich/6 männlich). Von den minderjährigen Patienten stammten 5 aus Kollektiv A und 6 aus Kollektiv B.

Hinsichtlich der Komorbiditäten lag bei insgesamt 87 Patienten (22 % [*n* = 87/396]) in der Anamnese und/oder oralen Provokation eine NSAID-Intoleranz vor. Bei 186 Patienten (41,5 % [*n* = 186/448]) bestand anamnestisch die Diagnose einer Typ-I-Allergie. Ein komorbides Asthma bronchiale war bei 167 der CRSwNP-Patienten (36,8 % [*n* = 167/454]) diagnostiziert.

### Klinischer Verlauf – vor Erstvorstellung

Der Großteil der Patienten (81,3 % [*n* = 340/418]) wurde durch den HNO-Facharzt an die Hochschulambulanz überwiesen.

Eine vorherige Behandlung mit nasalen Kortikosteroiden war anhand der Dokumentation bei 259 Patienten erfolgt (56,9 % [*n* = 259/455]), wobei eine klare Zunahme im Vergleich der Kollektive zu verzeichnen war: Bei Kollektiv A war bei 43,9 % [*n* = 108/246] der Patienten im Vorfeld eine Therapie mit nasalen Kortikosteroiden eingeleitet worden, gegenüber 72,2 % [*n* = 151/209] des Kollektivs B. Ein ähnliches Bild zeigt sich bei der Auswertung einer vorherigen Behandlung mit systemischen Steroiden: Bei Kollektiv A war bei 20 % [*n* = 49/245] der Patienten im Vorfeld eine Therapie mit systemischen Kortikosteroiden eingeleitet worden, gegenüber 45 % [*n* = 95/211] des Kollektivs B.

Bei 180 Patienten (39,8 % [*n* = 180/452]) war vor Erstvorstellung in der Hochschulambulanz mindestens eine NNH-Operation durchgeführt worden. Die Anzahl der vorherigen NNH-Operationen wird im folgenden Abschnitt in Abb. 4a zusammengefasst. Das Ausmaß der erfolgten Operation (alleinige Polypenentfernung vs. endoskopische Pansinus-Operation) war retrospektiv in den meisten Fällen nicht zu evaluieren. Im Mittel lag die vorherige Operation 8,6 Jahre vor Erstvorstellung zurück, mit einer Zeitspanne von < 1 Jahr bis 33 Jahre.

### Klinischer Verlauf – nach Erstvorstellung

Von allen 463 Patienten wurden 173 (37,4 %) nur einmal im Rahmen der Erstvorstellung in der Hochschulambulanz vorstellig, es erfolgte keine weitere ambulante Vorstellung im Zeitraum bis zur Erhebung der Daten.

**Abb. 2:**
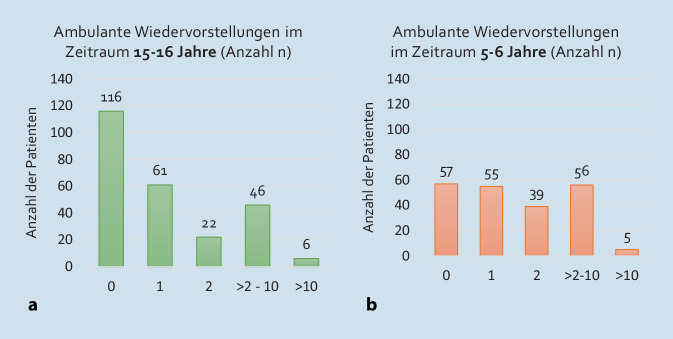
**a** Anzahl (*n*) der ambulanten Wiedervorstellungen der Patienten in der Hochschulambulanz nach Erstvorstellung in den Jahren 2007/08 (Kohorte A; Gesamtzahl der ausgewerteten Patienten: 251). **b** Anzahl (*n*) der ambulanten Wiedervorstellungen der Patienten in der Hochschulambulanz nach Erstvorstellung in den Jahren 2017/18 (Kohorte B; Gesamtzahl der ausgewerteten Patienten: 212)

Die Anzahl der ambulanten Wiedervorstellungen nach der Erstvorstellung in den Jahren 2007/08 (Kollektiv A) bzw. 2017/18 (Kollektiv B) vergleicht die Abb. 2a, b.

Eine stationäre Betreuung war im weiteren Verlauf aller 463 Patienten mindestens einmal bei 292 Patienten (63,1 %) erforderlich.

**Abb. 3:**
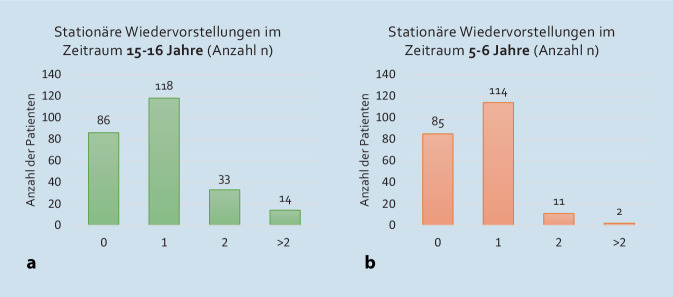
**a** Anzahl (*n*) der stationären Wiedervorstellungen der Patienten in der Hochschulambulanz nach Erstvorstellung in den Jahren 2007/08 (Kohorte A; Gesamtzahl der ausgewerteten Patienten: 251). **b** Anzahl (*n*) der stationären Wiedervorstellungen der Patienten in der Hochschulambulanz nach Erstvorstellung in den Jahren 2017/18 (Kohorte B; Gesamtzahl der ausgewerteten Patienten: 212)

Die Anzahl der stationären Wiedervorstellungen im Untersuchungszeitraum der Patientenkohorten 8 fassen die Abb. 3a, b zusammen.

Ursachen für eine stationäre Aufnahme waren bei der überwiegenden Anzahl der Patienten eine NNH-Operation (94,9 % [*n* = 277/292]) und/oder eine stationäre ASS-Provokation (15,1 % [*n* = 44/292]). In Einzelfällen kam es zum Erfordernis einer i.v.-Antibiose (2 Patienten), bei einem CRSwNP-Patienten erfolgte eine stationäre Überwachung bei anaphylaktischer Reaktion unter allergenspezifischer Immuntherapie.

**Abb. 4:**
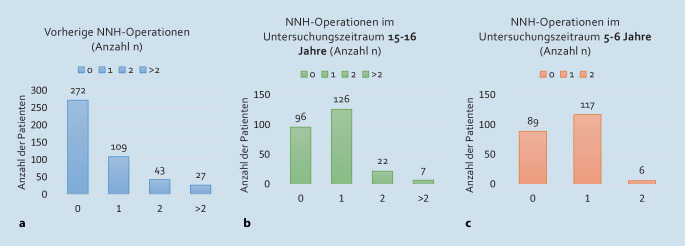
**a** Anzahl (*n*) der durchgeführten Nasennebenhöhlen(NNH)-Operationen vor Erstvorstellung in der Hochschulambulanz (Gesamtzahl der ausgewerteten Patienten: 452, 11 Patienten ohne Angabe). **b** Anzahl (*n*) der durchgeführten NNH-Operationen in der Universitätsklinik im Erhebungszeitraum nach Erstvorstellung in den Jahren 2007/08 (Kohorte A; Gesamtzahl der ausgewerteten Patienten: 251). **c** Anzahl (*n*) der durchgeführten NNH-Operationen in der Universitätsklinik im Erhebungszeitraum nach Erstvorstellung in der Hochschulambulanz nach Erstvorstellung in den Jahren 2017/18 (Kohorte B; Gesamtzahl der ausgewerteten Patienten: 212)

Außerdem wurde die Anzahl der durchgeführten NNH-Operationen nach Erstvorstellung in der Hochschulambulanz ausgewertet (Abb. 4b, c). Von Kollektiv A lag ein analysierter Zeitraum von 15–16 Jahren vor, von Kollektiv B 5–6 Jahre. Aus Kollektiv A konnten 88 % der Patienten sowie aus Kollektiv B 97 % der Patienten mit maximal einer NNH-Operation nach Erstvorstellung bislang therapiert werden.

#### Operationskomplikationen

Insgesamt kam es bei 13 der 278 operierten Patienten (4,7 %) zu einer Nachblutung. Wird die gesamte Anzahl der durchgeführten Operationen (*n* = 319) berechnet, handelt es sich um eine Nachblutungsrate von 4 %. In 12 Fällen war diese komplikations- und folgenlos zu therapieren, in einem Fall nach Revisionsoperation kam es aufgrund der Nachblutung zu mehrfachen erneuten Eingriffen und einem prolongierten stationären Aufenthalt mit anschließender Rehabilitationsmaßnahme. Bei 2 der 278 Patienten (0,7 %) musste die Operation im Rahmen der Narkoseeinleitung bzw. kurz nach Operationsbeginn aufgrund eines Bronchospasmus/einer anaphylaktischen Reaktion abgebrochen werden.

Insgesamt kam es bei 101 der 278 Patienten (36,3 %), die mindestens einmal eine NNH-Operation nach Erstvorstellung erhielten, zu einem Rezidiv der CRSwNP nach der ersten stattgehabten NNH-Operation im analysierten Zeitraum. In Kollektiv A wurde bei 57 der 155 Patienten (36,8 %) und in Kollektiv B bei 44 der 123 Patienten (35,8 %) ein Rezidiv der CRSwNP nach einmaliger Operation nach Erstvorstellung dokumentiert. Bei insgesamt 48 der 101 Rezidivpatienten (47,5 %) trat die erneute Polyposis innerhalb eines Jahres wieder auf, im Mittel kam es nach 2,4 Jahren zum Auftreten des Rezidivs.

#### NSAID-Intoleranz bzw. Asthma bronchiale

Ausgewertet wurde zudem das anamnestische Vorliegen einer NSAID-Intoleranz bzw. eines Asthma bronchiale bei Patienten mit einem Rezidiv der CRSwNP nach der ersten stattgehabten NNH-Operation.

In Kollektiv A gaben 30 von 57 Patienten (52,6 %) ein Asthma bronchiale an. Hinsichtlich der NSAID-Intoleranz lagen aufgrund des retrospektiven Settings bei 51 von 57 Patienten Angaben vor: Von den 51 Patienten wiesen 22 (43,1 %) eine NSAID-Intoleranz auf. Insgesamt bestand von den 51 Patienten bei 19 Patienten (37,3 %) eine NSAID-Intoleranz *und* ein Asthma bronchiale.

In Kollektiv B gaben 22 von 44 Patienten (50 %) ein Asthma bronchiale an. Hinsichtlich der NSAID-Intoleranz lagen aufgrund des retrospektiven Settings bei 41 von 44 Patienten Angaben vor: Von den 41 Patienten bestand bei 13 (31,7 %) eine NSAID-Intoleranz. Insgesamt wiesen von den 41 Patienten 10 Patienten (24,4 %) eine NSAID-Intoleranz *und* ein Asthma bronchiale auf.

#### ASS-Provokation/-Desaktivierung

Eine stationäre Aufnahme zur überwachten Provokation und ggf. Desaktivierung mit ASS erfolgte insgesamt bei 44 Patienten (9,5 %). Davon waren 35 Patienten aus der Kollektiv A, 9 CRSwNP-Patienten wurden aus Kollektiv B zur stationären ASS-Provokation/-Desaktivierung aufgenommen. Bei 32 Patienten, die unter einer ASS-Erhaltungstherapie nach hausinterner Empfehlung mit 300 mg ASS protect täglich standen, lagen zum Zeitpunkt der Auswertung Nachbeobachtungsdaten über die weitere ASS-Einnahme vor. Bei 12 dieser 32 Patienten (38 %) war inzwischen ein Abbruch der ASS-Dauertherapie dokumentiert, nach einem Mittelwert der Therapieeinnahme von 1,6 Jahren. Die am häufigsten genannten Gründe für den Therapieabbruch waren unzureichende Befundbesserung, Blutungskomplikationen, geplante elektive Operationen und gastrointestinale Nebenwirkungen.

#### Antikörpertherapie

Insgesamt erhielten 16 der 463 Patienten (3,5 %) eine Biologikatherapie aufgrund eines protrahierten Verlaufs der CRSwNP. Jeweils 8 Patienten stammten aus Kollektiv A und 8 Patienten aus Kollektiv B. Zum Zeitpunkt der Auswertung waren 9 der 16 Patienten unter einer Therapie mit Dupilumab 300 mg subkutan, 3 der 16 Patienten wurden mit Omalizumab (nach Gesamt-IgE/Körpergewicht) therapiert und 2 Patienten mit Mepolizumab 150 mg. Bei 2 der 16 Patienten war im Verlauf ein Wechsel des Antikörpers vorgenommen worden.

## Diskussion

Die CRSwNP als multifaktorielle inflammatorische Erkrankung kann für die betroffenen Patienten eine signifikante Belastung im Alltag werden. Grund sind u. a. die belastenden Symptome wie Riechstörungen, nasale Obstruktion und Rhinorrhö, sowie die Rezidivtendenz und Komorbiditäten wie das Asthma bronchiale und NSAID-Intoleranz. Nicht zu unterschätzen ist die Belastung der Gesellschaft durch häufig erforderliche Inanspruchnahme von Gesundheitseinrichtungen, Operationen mit zunehmendem Risiko und Arbeitsausfall aufgrund der chronischen Erkrankung [14]. Die Therapiemöglichkeiten sind vielfältig und aktuell im Wandel durch die zunehmende Etablierung der Biologikagabe bei refraktären Verläufen. Letztendlich muss die Antikörpertherapie unter Berücksichtigung des Wirtschaftlichkeitsgebots für Biologikaverordnungen im deutschen GKV-System erfolgen: ausreichend, zweckmäßig, notwendig. Die Wirtschaftlichkeit ergibt sich hierbei aus dem Nutzen der Maßnahme in Relation zu den dafür eingesetzten Aufwendungen, insbesondere den Kosten [14].

Von der Gesamtzahl der 463 analysierten Patienten wurden 173 (37,4 %) nur einmal im Rahmen der Erstvorstellung in der Hochschulambulanz vorstellig, in diesem Rahmen wurde zunächst ein konservatives Vorgehen besprochen, je nach Vortherapie und Befund mit lokalen Kortikosteroiden mit oder ohne zusätzliche systemische Steroidgabe. Es ist zu diskutieren, dass bei einzelnen Patienten die Ergebnisse nicht repräsentativ sind, da beispielsweise eine andere Klinik konsultiert wurde und somit die weiterhin erforderliche Behandlung nicht erfasst wurde. Dies ist eine Limitation der vorliegenden Arbeit, die bei der Interpretation der Ergebnisse berücksichtigt werden muss. Dasselbe gilt für das Ergebnis, dass es insgesamt bei 101 Patienten (36,3 %) zu einem Rezidiv der CRSwNP nach der ersten stattgehabten NNH-Operation im analysierten Zeitraum nach Erstvorstellung in der Hochschulambulanz kam. Da bei den Patienten aus Kollektiv A ein Zeitraum von 15–16 Jahren überblickt werden konnte, kann dies dennoch darauf hinweisen, dass ein Anteil der Patienten von einer NNH-Operation (FESS) über längere Zeit profitiert und diese somit bei geringem Operations- und Narkoserisiko weiterhin einen sinnvollen ersten Therapieschritt darstellt. Diskussion über diese Aussage liefert u. a. die Indikationsstellung der Biologika, in welcher je nach Präparat die Zulassung als Add-on-Therapie für CRSwNP-Patienten besteht, die mit systemischen Kortikosteroiden *und/oder* chirurgischem Eingriff nicht ausreichend kontrolliert sind [19]. Eine vorangehende Operation ist somit rein aufgrund der Zulassung nicht zwingend gefordert. Ein weiterer wichtiger Faktor ist, dass das Ausmaß und die Qualität einer NNH-Operation deutlich variieren kann und somit in die individuelle Therapieentscheidung mit einfließen sollte. Eine Schwierigkeit stellt hierbei dar, dass bezüglich der Beurteilung der genannten Faktoren keine einheitliche oder standardisierte Vorgehensweise vorhanden ist. Zudem steht weiterhin zur Diskussion, ob eine umfangreichere Operation zu einem besseren postoperativen Verlauf bzw. einer geringeren Rezidivrate führt.

Basis für eine Reduktion von Komplikationen bei der FESS ist das prä- und perioperative Setting: Hierzu gehört eine präoperative Schichtbildgebung bei allen Patienten mit Berücksichtigung der anatomischen Gegebenheiten durch den Operateur, die Operation mit Navigation insbesondere bei fehlenden Landmarken nach Voroperation und ein möglichst blutungsarmes Prozedere, z. B. durch engmaschige perioperative Blutdruckeinstellung [7, 16, 17]. Nichtsdestotrotz können nach NNH-Operationen prinzipiell auch schwere Komplikationen auftreten. In einer retrospektiven Kohortenanalyse mit fast 80.000 Patienten nach FESS fanden Krings et al. eine Rate an schwerwiegenden Komplikationen von circa 0,4 %. Bei älteren Patienten und bei Fällen mit mehrfachen NNH-Voroperationen war die Wahrscheinlichkeit für eine relevante Komplikation im Vergleich erhöht [13]. Dennoch ist eine Frage, die sich gerade nach dem Auftreten einer schweren Komplikation stellt, inwieweit prinzipiell alle CRSwNP-Patienten präoperativ über die Alternative einer Biologikatherapie beraten werden sollten. Bei fehlender Langzeiterfahrung mit den Antikörpern in dieser Indikation sowie unter Berücksichtigung des Wirtschaftlichkeitsgebots ist dies nach Einschätzung der Autoren eine Individualentscheidung, in die auch das Narkoserisiko und Operationsrisiko (z. B. anatomische Begebenheiten) mit einbezogen werden sollten.

Die Ergebnisse können unter Berücksichtigung der genannten Limitationen darauf hinweisen, dass ein relevanter Anteil der CRSwNP-Patienten ausschließlich durch eine konservative Therapie über Jahre ausreichend behandelt werden kann. Eine stationäre Betreuung war im Verlauf aller 463 Patienten mindestens einmal bei 292 Patienten (63 %) erforderlich, im Umkehrschluss wurden knapp 40 % der Patienten rein ambulant konservativ therapiert. Hier ist zu beachten, dass schon eine Vorselektion erfolgte, da die Patienten i. d. R. durch den HNO-Facharzt oder Allgemeinarzt überwiesen wurden. Dass sich das stufenweise Vorgehen mit nasalen Steroiden im Laufe der Jahre zunehmend etabliert hat, zeigt das Ergebnis, dass von der Patientenkohorte A 43,9 % der Patienten im Vorfeld eine Therapie mit nasalen Kortikosteroiden hatte, gegenüber 72,2 % der Patienten des Kollektivs B. Ein vergleichbares Bild zeigte sich bei der Auswertung einer vorherigen Behandlung mit systemischen Steroiden. (Kollektiv A: 20 % der Patienten, gegenüber 45 % des Kollektivs B). Ein unsicherer Faktor bleibt dabei insbesondere bei den topischen Steroiden die Compliance einer regelmäßigen Einnahme [9].

Zum aktuellen Zeitpunkt sind die 3 zugelassenen Antikörper ausschließlich für Erwachsene in der Indikation CRSwNP zugelassen, in anderen Indikationen sind dieselben Präparate schon in deutlich jüngerem Alter zugelassen (z. B. Dupilumab bei der eosinophilen Ösophagitis oder atopischen Dermatitis) [18]. Die CRSwNP ist zwar vorwiegend eine Erkrankung des mittleren Alters, 10 Patienten der vorliegenden Studie waren zum Zeitpunkt der Erstvorstellung in der Ambulanz der Autoren jedoch minderjährig. In Einzelfällen kann bei protrahierten Verläufen eine Antikörpertherapie auch vor dem Erwachsenenalter erforderlich sein.

Insgesamt zeigen die vorliegenden Daten, dass die Anzahl der Patienten zur ASS-Provokation und adaptiven Desaktivierung mit ASS abnimmt. Die genaue Abbruchrate der sog. ASS-Erhaltungstherapie in der vorliegenden Untersuchung beinhaltete aufgrund des retrospektiven Settings eine Dunkelziffer, Nebenwirkungen der Therapie waren aber häufig und alltagsrelevant. Diese Ergebnisse decken sich mit Ergebnissen der Literatur [5, 11]. Hinsichtlich des zukünftigen Stellenwerts der ASS-Desaktivierung in Deutschland verbleibt es, auf die Empfehlung der überarbeiteten S2k-Leitlinie der AWMF zu warten.

Die relativ geringe Anzahl von Patienten, die eine Antikörpertherapie erhielten (16 der 463 Patienten; 3,5 %), in der vorliegenden retrospektiven Arbeit zeigt, dass unter genauer Indikationsstellung gemäß den aktuellen Positionspapieren, Empfehlungen und Leitlinien ein wirtschaftliches Vorgehen möglich ist. Wie schon genannt ist das Fehlen von Langzeitergebnissen und das Vorliegen weiterer offener Fragen (z. B. der Wechsel unter den 3 zugelassenen Präparaten im Individualfall) ein Ausblick in die Zukunft, in welcher Patientenregister und randomisiert kontrolliert durchgeführte Studien weitere Ergebnisse zum therapeutischen Vorgehen bei CRSwNP-Patienten bringen können.

Eine weitere Limitation der vorliegenden Studie ist das retrospektive Setting, welches allerdings die große Patientenzahl ermöglichte. Hierdurch waren einzelne Ergebnisse nur in ihrer Tendenz zu bewerten (beispielsweise die Abbruchrate unter den Patienten mit empfohlener ASS-Erhaltungstherapie), da nicht alle Patienten in der Universitätsklinik wieder vorstellig wurden. Auch durch Umzug oder Klinikwechsel ist die Auswertung möglicherweise beeinflusst.

## Fazit für die Praxis

- Die 3 zugelassenen Antikörper Dupilumab, Omalizumab und Mepolizumab bieten eine neuartige Behandlungsmöglichkeit für Patienten mit therapierefraktärer chronischer Rhinosinusitis mit Polyposis nasi (CRSwNP).
- Aufgrund der Abbruch- und Nebenwirkungsrate der Patienten unter Acetylsalicylsäure(ASS)-Erhaltungstherapie ist zu erwarten, dass perspektivisch die Antikörpertherapie die Indikation zur ASS-Dauereinnahme signifikant reduzieren wird.
- In der Regel ist die CRSwNP eine Erkrankung des mittleren Lebensalters.
- Selten sind jedoch auch Minderjährige betroffen, bei denen die Antikörperbehandlung (bislang) noch nicht zugelassen ist.
